# Sperm granulomas: Predictive factors and impacts on patency post vasectomy reversal

**DOI:** 10.1111/and.14439

**Published:** 2022-05-06

**Authors:** Mary K. Samplaski, John C. S. Rodman, Jessica Michelle Perry, Matthew B. F. Marks, Robert Zollman, Kian Asanad, Sheldon F. Marks

**Affiliations:** ^1^ Institute of Urology University of Southern California Los Angeles California USA; ^2^ Southern California Clinical and Translational Science Institute University of Southern California Los Angeles California USA; ^3^ International Center for Vasectomy Reversal Tucson Arizona USA

**Keywords:** granuloma, tobacco, vasectomy reversal, vasoepididymostomy, vasovasostomy

## Abstract

The objective of this study was to identify factors that predict for sperm granuloma formation and the impact of sperm granuloma presence and quantity on vasectomy reversal (VR) outcomes. A cross sectional retrospective review of prospectively collected data, on the impact of granuloma on VR outcomes from a single academic center was performed. The impact of age, obstructive interval, intraoperative vasal fluid findings, anastomosis type, body mass index, tobacco use and total motile count (TMC) was determined. A total of 1550 men underwent VR between January 2000 and August 2019. Granulomas were present unilaterally in 23.3% (*n* = 361) and bilaterally in 14.2% (*n* = 220). On univariate analysis, increasing patient age negatively correlated with a larger number of granulomas (*p* = .011). Granuloma presence was associated with finding intact and motile sperm from the vasal stump intraoperatively (*p* = .001), and vasoepididymostomy anastomosis (*p* < .001). However, granuloma presence (and quantity) did not correlate with obstructive interval or maximum TMC. Tobacco use and body mass index (BMI) were not associated with granuloma presence. On multivariate analysis, granuloma quantity was not associated with TMC. Obstructive interval and vasovasostomy anastomosis were associated with higher TMC, while BMI was negatively associated with TMC. In conclusion, increasing age was negatively correlated with granuloma formation. Granuloma presence was associated with more favourable intraoperative fluid findings and anastomosis type, but not post‐VR TMC, suggesting men with and without granulomas undergoing skilled microsurgery will have similar patency rates. Heavier men should be encouraged for weight loss prior to vasectomy reversal as increasing BMI was associated with lower TMC.

## 1 INTRODUCTION

Vasectomy is the only FDA approved method of male contraception, and is widely considered to be the safest option for permanent sterilization, with long‐term failure rates of less than 1% (Schwingl & Guess, 2000). It is estimated that 5%–6% of men undergoing vasectomy will desire reversal for restoration of fertility due to a change in marital status or reproductive goals (Masterson et al., 2013; Potts et al., 1999). With advances in, and growing acceptance of, assisted reproductive technologies, couples have more treatment options of fertility available. The importance of identifying factors that may predict success after vasectomy reversal (VR) is paramount for men, in order that they can best choose among their reproductive options.

During a VR, the male reproductive anatomy is restored to continuity, either by vasovasostomy or vasoepididymostomy. Patency and post‐surgical reproductive outcomes, vary greatly after VR with published patency rates ranging from 62%–97% and pregnancy rates ranging from 26%–92% (Schwingl & Guess, 2000; Sharlip, 1993; Silber & Grotjan, 2004). The Vasovasostomy Study Group (Belker et al., 1992) identified several factors as correlating with post‐reversal patency, one of which was the presence of granuloma. These are seen in approximately 60% of males post VR (McDonald, 1996).

Sperm granulomas are the body's reactive response to the leakage of sperm. Following vasectomy, the testicle continues to produce sperm, and their presence in the obstructed vas raises the intraluminal pressure (McDonald, 1996). Animal data has shown that intraluminal pressures post‐vasectomy are highest in the cauda epididymis and vasal stump (McDonald, 1996). These lesions are composed of sperm, macrophages and other immune cells involved in the inflammatory response, which develop into a small mass on the testicular side of the detached vas deferens, often surrounded by varying degrees of scar tissue. Granulomas form when sperm leak out of the vasal stump, a reaction to the back‐pressure from congestion in the obstructed vas deferens (Shapiro & Silber, 1979; Silber, 1977a). This leakage of sperm is suspected to allow for the continuous production of sperm in a low‐pressure state, which has been one of the proposed mechanisms for increased fertility in post VR men with sperm granulomas (Shapiro & Silber, 1979; Silber, 1977a).

Since then there have been conflicting data on the impact of granuloma presence on VR success rates. A study of 213 VRs by a single surgeon found that men with at least a unilateral sperm granuloma had patency of 95% versus 78% without granulomas, which did not reach statistical significance (*p* = .07). No difference in pregnancy rate with or without granulomas were seen (Boorjian et al., 2004). A different series of 334 VRs by a different single surgeon, found that the presence of a granuloma did not prognosticate for post‐operative patency or pregnancy rates (Magheli et al., 2010). Finally, a predictive nomogram predicting patency after VR did identify the presence of granuloma as a predictive factor (Hsiao et al., 2012).

At present, the factors known to impact post‐VR success include, vasal obstructive interval, vasal fluid quality, the presence of sperm in the vasal fluid, microsurgical technique and postsurgical care (Belker et al., 1992; Hsiao et al., 2012; Namekawa et al., 2018). The prior series have yielded conflicting results and are limited by small sample sizes. Better understanding of the impact of granuloma presence on VR outcomes could help prognosticate for patients, which could in turn help with decision making for VR versus surgical sperm retrieval.

In this study, we investigate what factors are correlated with granuloma formation, including obstructive interval, age, body mass index (BMI) and tobacco use. We also seek to understand the impact of sperm granuloma presence and quantity on factors known to impact post‐VR total motile sperm count (TMC), including intraoperative vasal fluid composition, obstructive interval, male age and anastomosis type.

## 2 MATERIALS AND METHODS

Institutional review board approval was obtained. A retrospective review of VRs performed between January 2000 and August 2019 by one of two high‐volume microsurgeons (SFM or PJB), at a single institution. All VR were being performed for fertility restoration. Men were advised to undergo post‐VR semen analysis (SA) within four months of VR. Men were excluded if they did not have postoperative SA testing or were using exogenous testosterone.

All vasovasostomies (VVs) were performed using a mini‐incision two‐layer microsurgical technique, with six equally spaced 10.0 nylon sutures on the mucosa, and six equally spaced 9.0 nylon sutures on the muscularis. All vasoepididymostomies (VEs) were performed using the end‐to‐side intussusception technique, using four equally spaced 10.0 nylon sutures on the mucosa and four 9.0 nylon sutures on the muscularis. VV was performed if the vasal stump fluid was clear, thin, copious and if sperm or sperm parts were seen at intraoperative bench microscopic evaluation. VE was performed if the fluid obtained was pasty, thick and no sperm or sperm parts were seen.

The impact of one or more sperm granulomas on post‐VR outcomes were analysed, including male age, anastomosis type (bilateral VV, bilateral VE, or unilateral VV and unilateral VE), post‐VR TMC and the vasal obstructive interval. The impact of patient factors on granuloma presence and quantity was also analysed, including age, patient‐reported tobacco usage (cigarettes, chew, marijuana), and BMI.

Descriptive statistics were presented using median (IQR) or mean (SD), dependent upon distribution, for continuous variables, and frequency (percent) for categorical. Two‐sample *t*‐test and one‐way ANOVA, or their non‐parametric equivalent, were used to test for differences in continuous variables and Pearson's chi‐squared or Fisher's Exact test were used to test for associations in categorical variables. Multivariable logistic and ordinal logistic regression were used to evaluate the associations between independent variables of interest with granuloma presence, while controlling for patient age. Multivariable negative binomial regression was performed to evaluate the association between independent variables of interest and maximum TMC, while controlling for age. All statistical analyses were performed using R version 4.0.2. All tests were two‐sided and a *p*‐value <.05 was considered statistically significant.

## 3 RESULTS

During the study period, 4817 men underwent VR, and after excluding those that did not meet criteria, 1550 were included in analysis. The mean ± SD male age was 39.9 ± 6.8 years. The mean ± SD male BMI was 27.9 ± 4.8. The mean obstructive interval was 8.6 ± 5.6 years. The majority, 1466 (94.6%) of men underwent VV/VV, 71 (4.6%) underwent VV/VE and 13 (0.8%) underwent VE/VE. The mean post‐VR TMC was 58.2 ± 79.0 × 10^6^ sperm.

Univariate analysis of the impact of granuloma presence and quantity is summarized in Table 1. Patient age was negatively associated with the number of granulomas seen (*p*‐.011). Patients with two granulomas were younger than patients with zero or one granuloma. Granulomas were present on one side in 23.3% (*n* = 361) and both sides in 14.2% (*n* = 220). Granuloma presence was associated with intraoperative vasal fluid findings, including the presence of both complete motile sperm (*p* = .001) and complete non‐motile sperm (*p* = .001). Likewise, the presence of granuloma was associated with the type of anastomosis performed (VV or VE), with granuloma presence being more likely to undergo VV anastomosis. However, the presence of granuloma did not correlate with post‐VR maximum TMC or obstructive interval.

**TABLE 1:** Impact of granuloma presence and quantity on vasectomy reversal outcomes

Variable	*N*	Number of granulomas (median ± IQR, or n [%])^a^			*p*‐value
		0 (*n* = 969)	1 (*n* = 361)	2 (*n* = 220)	
Maximum total motile count (million sperm/ml)	1550	32.4 ± 71.2	34.0 ± 68.1	31.7 ± 74.1	.682
Patient age (years)	1548	39.0 ± 9	39.0 ± 9	38.0 ± 8	.011*
Obstructive interval (years)	1547	8.0 ± 8	8.0 ± 8	9.0 ± 7.3	.348
*Anastomosis type*					<.001*
VE/VE	13	12 (1.2%)	1 (0.3%)	0	
VV/VV	1466	901 (93.0%)	345 (95.6%)	220 (100%)	
VV/VE	71	56 (5.8%)	15 (4.2%)	0	
*Smoking/Tobacco use*					.207
No	1277	803 (84.3%)	286 (80.8%)	188 (85.8%)	
Yes	249	150 (15.7%)	68 (19.2%)	31 (14.2%)	

^a^ Numbers represent Median (IQR) (min, max) for continuous variables and frequency (column percent) for categorical.

* Significant at *p* = .05 (Fisher's Exact or independent *T*‐test).

A multivariate negative binomial regression, with TMC as the primary outcome was performed. After adjusting for age and smoking status, number of granulomas was not associated with TMC (Table 2). BMI was significantly negatively associated with TMC. For every unit increase in BMI there was a 2% decrease in the TMC (IRR [95%CI] = 0.98 [0.96–0.99]; *p* = .005). Obstructive interval was significantly negatively associated with maximum TMC. For every year increase in obstructive interval there was a 4% decrease in TMC (IRR [95%CI] = 0.96 [0.95–0.98]; *p* < .001). Anastomosis type was also significantly associated with TMC. Patients that had VE had, on average, a 71% lower TMC when compared to patients that had VV anastomosis (IRR [95%CI] = 0.29 [0.12–0.67]; *p* = .004).

**TABLE 2:** Multivariable negative binomial regression with total motile count as outcome

Variable	Coefficient	IRR (95% CI)	*p*‐value
*Number of granulomas*			
0	Reference	Reference	
1	0.032	1.03 (0.86–1.24)	.730
2	0.108	1.11 (0.90–1.38)	.315
Patient age (years)	−0.004	1.00 (0.98–1.01)	.521
BMI	−0.023	0.98 (0.96–0.99)	.005*
Obstructive interval (years)	−0.038	0.96 (0.95–0.98)	<.001*
*Smoking/Tobacco use*			
No	Reference	Reference	
Yes	−0.077	0.93 (0.76–1.13)	.455
*Anastomosis type*			
VV/VV	Reference	Reference	
VE/VE	−1.244	0.29 (0.12–0.67)	.004*
VV/VE	−0.264	0.77 (0.53–1.12)	.169

* Significant at *p* = .05 level.

Abbreviation: IRR, incidence rate ratio.

## 4 DISCUSSION

The impact of granuloma presence on post VR patency and pregnancy rates remains unclear. Prior series have been limited by patient number, as well as data on anastomosis type. In addition, all prior series report the presence of granuloma, but do not record laterality, or correlate this with the laterality of intraoperative vasal fluid quality, or anastomosis type. Our series represent the largest series of men undergoing VR for restoration of fertility, nearly 5000 men, of which nearly one‐fourth had granulomas.

Prior data has shown that granuloma presence has been associated with better quality intraoperative vasal fluid (Belker et al., 1983; Silber, 1977a), thought to be from a “pop‐off valve” pressure releasing effect (Witt et al., 1994). Interestingly, ours is the first study to look at the relationship between intraoperative vasal fluid findings and the presence of granuloma. All prior studies look at the presence of granuloma and anastomosis type, without reporting the intraoperative fluid findings. We observed this “pop‐off valve” effect in our data, as granuloma presence was associated with more favourable intraoperative vasal fluid findings, including the presence of both complete motile sperm and complete non‐motile sperm. In a Cochrane Database Systematic Review literature evaluating predictors of granuloma formation in terms of vasectomy technique demonstrated there was no difference with vasectomy with clips versus conventional vasectomy, ligation and excision with or without fascial interposition, and intra‐vas device versus no‐scalpel vasectomy (Cook et al., 2014).

Likewise, we did not find that granuloma presence correlated with vasal obstructive interval. We are the first group to make this association as well, between vasal obstructive interval and granuloma presence. It seems that patients will either form granulomas or they will not, but that the timeline does not matter as much. Patient age was associated with the number of granulomas seen, consistent with the prior literature (Magheli et al., 2010). In future studies, it would be interesting to evaluate the vasal stump length and determine if this impacted granuloma presence, as this has not been looked in the prior literature.

The prior literature is conflicting is on the effect of granuloma on post VR semen parameters or patency. A 2009 retrospective review of 351 VR cases found that the presence of a sperm granuloma was predictive of surgical patency rates (Hinz et al., 2009). Other earlier series have corroborated this, finding that granuloma presence correlated with higher post VR semen parameters (Belker et al., 1983; Bolduc et al., 2007; Fenig et al., 2012; Silber, 1977b). However, the Vasovasostomy Study Group, and others have found no impact of granuloma on patency or fertility rates (Belker et al., 1992; Boorjian et al., 2004; Lee, 1986). We found that granuloma presence did not correlate with post‐VR maximum TMC.

Connecting the intraoperative vasal fluid quality and post VR semen parameters, we found that granuloma presence was associated with anastomosis type, with granuloma presence being more likely to undergo VV anastomosis. This is similar to a 2012 study that found that the presence of granuloma correlated with a lower need for VE (Fenig et al., 2012). In order words, this suggests that a man >15 years after vasectomy with a granuloma has a higher VV anastomosis rate compared to a man >15 years without a granuloma, and thus higher success rates. There is an abundance of data showing that microsurgeons who perform VR should be able to perform VE, a more technically challenging operation than VV (Chawla et al., 2004). In some cases, VE anastomoses may yield lower postoperative sperm counts when compared with VVs (Schwarzer & Steinfatt, 2014). This may explain part of the conflicting association seen between granulomas and post VR semen parameters, since many of the prior studies do not include data on VE versus VV anastomosis.

We found that for a single, high‐volume VR microsurgeon, skilled in doing both VV and VE, the post‐VR TMC was the same both with and without unilateral and bilateral granulomas. These data show that in skilled hands, proficient in performing VE and VV, good microsurgery is effective at restoring sperm to the ejaculate for men undergoing VR.

Additionally, this is the first study to study the effect of tobacco use and BMI on granuloma presence. There are some studies looking at BMI and varicocele, the question being if higher BMI results in higher pressures in the pampiniform plexus, resulting in varicocele formation. However, a recent meta‐analysis found that larger BMI was actually protective against varicocele presence (Xiao‐Bin et al., 2021). This is the first series to look at the effect of BMI on granuloma presence. Intuitively, higher BMI would have the potential to increase pressures transmitted to the testicle. However, our data show that it does not impact granuloma formation. Interestingly, while BMI did not impact granuloma presence, it did negatively correlate with TMC. For every unit increase in BMI there was a 2% decrease in the TMC.

Finally, there is some literature analysing the effect of tobacco use on VR outcomes. While some data does show that tobacco use decreased post‐VR pregnancy rates (Nusbaum et al., 2020), although other data did not identify this correlation (van Dongen et al., 2012). None of the available studies included smoking status and the presence of granuloma. We found that patient reported usage of tobacco (cigarettes, chew, marijuana) was not associated with unilateral or bilateral granuloma presence, or with post‐VR TMC.

Our study does have limitations. Our sperm granulomas were not sent for pathologic evaluation to determine if suture or sperm granuloma was present. However, this was also not performed in most of the prior published series looking at this outcome (Belker et al., 1992; Boorjian et al., 2004), and our granuloma rate of 23% was consistent with the prior published literature on granuloma presence, which shows that 30% of VR cases will have at least one granuloma (Belker et al., 1992; Silber, 1977b).

## 5 CONCLUSIONS

This is the largest series evaluating the impact of granuloma presence on VR outcomes, and the first study to evaluate the impact of granuloma quantity. This is the first study demonstrating factors that significantly associate with granuloma presence. Granuloma presence was associated more favourable intraoperative fluid findings and anastomosis type, but not with post‐VR TMC. This would suggest that in skilled microsurgical hands, post‐VR TMC for VV and VE is similar, regardless of the presence of a granuloma. Tobacco use and heavier BMI were not associated with granuloma presence. However, higher BMI was correlated with a lower post‐VR TMC, and men with higher BMI should be encouraged for weight loss prior to VR.

## CONFLICT OF INTEREST

The author declares that there is no conflict of interest that could be perceived as prejudicing the impartiality of the research reported.

## ETHICAL APPROVAL

The present study protocol was reviewed and approved by the institutional review board of the University of Southern California (HS‐18‐00977).

## Data Availability

Data sharing is not applicable to this article as no new data were created or analyzed in this study.
